# Transcendental model selection: a computational account of symbolic cognition and general intelligence through morality and culture

**DOI:** 10.3389/fsoc.2026.1646503

**Published:** 2026-04-22

**Authors:** Shagor Rahman, Andrew Pashea

**Affiliations:** 1 Active Inference Institute, Westfield, NJ, United States; 2 Active Inference Institute, Chicago, IL, United States

**Keywords:** active inference, artificial intelligence, agent-based modeling, cultural evolution, moral psychology, structure learning, social cognition, symbolic communication

## Abstract

General intelligence enables flexible problem solving across diverse contexts by minimizing uncertainty. Symbolic systems such as language extend this capacity, allowing humans to build social groups and construct world models beyond typical biological constraints. Previous research on linguistic communication within active inference has emphasized deep hierarchical models that ensure shared semantics between communicators. We argue that these models, while powerful, require extension to account for symbolic genesis, specifically using morality not only as uncertainty minimization across cultural niches, but also as the mechanism that created the virtual space enabling symbolic cognition. Our ancestors transcended dyadic modeling by implementing cultural layers through novel model selection, enabling in-group signaling and hierarchical social organization through psychological typing. This rendered the generative process endogenous (self-referential). Our emotional and impulsive tendency toward morality, we argue, enabled the deeper level of abstraction and the stable third-party triangulated perspective necessary for symbolic thought. This framework can be evaluated through simulations similar to recent active inference literature and provides a foundation for building generally intelligent systems aligned with human cultural values.

## 1 Introduction

*What to others a trifle appears*

*Fills me full of smiles or tears;*

*For double the vision my eyes do see*,

*And a double vision is always with me*.

*With my inward eye, 'tis an old Man grey;*

*With my outward, a Thistle across my way*.

....

*Now I a fourfold vision see*,

*And a fourfold vision is given to me;*

'*Tis fourfold in my supreme delight*

– William Blake, *Letter to Thomas Butts* (1802) and *Milton* (1804–1810)

William Blake understood something central to the human experience and existence writ large. Beyond the myriad of perspectives that variously produce trifles and joys, it is our ability to traverse these various points of view and integrate them into our identity, achieving a greater depth of awareness, that constitutes our humanity.

This viewpoint captures a central challenge in the development of artificial general intelligence (AGI). How to create systems that match human cognitive flexibility while remaining aligned with human values? Current AI architectures, despite remarkable capabilities in narrow domains, lack the biological mechanisms that enable humans to seamlessly navigate between self-interest and social coordination, between immediate goals and cultural norms, between concrete reasoning and abstract symbolism (Sloman et al., 2021).

### 1.1 Multiple scales of humanity's vision and mind

Human cognition permits the ability to operate swiftly at multiple scales, as evidenced and afforded by language and other faculties. Central to our ability to toggle between multiple scales is our ability to inhabit a variety of social contexts that permit deep cultural learning, or information processing over vast spatiotemporal bounds. We routinely switch between optimizing personal outcomes, maintaining relationships, following social conventions, and upholding abstract principles. This flexibility is precisely what enables both individual creativity and collective coordination.

How can we represent the nature of general intelligence in computational terms? How can we best take at least inspiration from humanity's particular trajectory to symbolic and general intelligence? Active inference represents any entity's ability to solve problems across contexts as aligned with the hierarchical integration of information processing and uncertainty minimization across multiple scales.

### 1.2 In the beginning was the word

We present a computational account of the myth of objectivity hypothesis (Rahman, 2023), the view that the codification of behavioral norms is employed to signal in-group identity or morality. We name this transcendental model selection and inference to describe the ability to signal anonymous in-group identity that constitutes a cultural niche and how that depth is governed, respectively.

In order to understand words, phrases, grammatical constructions, and even complex mathematical notation, people adopt a shared perspective among communicators. This shared meaning necessarily exists beyond the individuals active within a given exchange. Language and all symbolic expression require the adoption of a cultural perspective. We must believe that symbols carry meaning beyond individual, dyadic, or social perspectives and are encoded in a virtual space (Chandler, 2022). This enables not only symbolic communication but also deep planning, allowing us to evaluate alternative policies, i.e., courses of action.

We propose that this cultural perspective, in line with the myth of objectivity, is developmentally and historically related to morality, specifically the codification of universal norms and the abstract social typing that occurs in relation to them. We formalize this relationship by offering a novel hierarchical implementation of active inference, conditioned on a type of model selection, i.e., structural learning. The former draws on prior studies in active inference that demonstrates the construction of deep and globally expansive predictive hierarchies (Sandved-Smith et al., 2021), particularly those addressing linguistic communication, while the latter, we believe, lacks precedent but fits into the mathematical arena of active inference, namely, as structured learning that features prominently within Bayesian inference.

### 1.3 Transcending standard inference

Generally, linguistic communication is described in active inference formulations as involving a deep hierarchy that allows for semantics, narrative, and concepts at deeper levels to guide things like syntax and lexical selection (e.g., word choice) at lower levels. We argue that a cultural level must subordinate existing semantics and introduce “transcendental inference,” an implementation of the predictive hierarchy within the free energy principle. It aims to explain our ability to integrate various levels of social interactions into a single cultural hierarchy. This integration is conditioned on a precision parameter, featured previously throughout active inference (Laukkonen et al., 2025), that subordinates interactions and motivations that occur at more superficial levels, including individual, dyadic, and anonymous tag-based social strata.

This model relies on a form of selection that allowed our ancestors to differentiate between agents faithful to cultural norms and deviants, suggesting that they inhabit a narrower, more egotistical model. This model selection constitutes a degree of structural learning, answering how an agent's generated observations minimize uncertainty and therefore indicate allegiance to deeper action sequences. This process is critical for maintaining sets of higher-order complexity models.

A puzzle emerges from decades of sociolinguistic research that existing computational models of language fail to address. From six months of age, infants prefer speakers with familiar accents (Kinzler, 2021), a bias with evolutionary roots that persists into adulthood. William Labov's foundational study demonstrated that linguistic variation reliably maps to socioeconomic stratification across New York City; subsequent research has confirmed this pattern across cultures (Bell et al., 2016; Eckert, 2012). Code-switching, the practice of adjusting language to fit context, functions as a strategic tool for navigating and negotiating identities (Kipchoge, 2024).

These findings reveal that language is not merely a tool for conveying information but a marker of identity deeply tied to social belonging. Yet prior active inference approaches to linguistic communication emphasize semantic-syntactic distinctions based on emergent joint models (Friston et al., 2020, 2024). Shared meaning, while necessary, cannot explain language's connection to identity, its role in signaling group membership, or the innate bias toward familiar linguistic markers that appears before semantic competence develops.

We argue that this gap reflects a deeper omission, i.e., the failure to recognize morality as constitutive of symbolic cognition itself. Tying language first to morality ties both to cognitive identity, making moral capacity core to active inference. Morality, on this view, is not primarily about cooperation or norm enforcement but about producing stable identity within an interconnected context.

Capacity created the cognitive architecture enabling symbolic thought. Our ancestors transcended dyadic modeling by implementing cultural layers through novel model selection, enabling in-group signaling and hierarchical social organization through psychological typing. This rendered the generative process endogenous (self-referential), creating the virtual space necessary for symbolic cognition.

What follows is a computational account of the myth of objectivity hypothesis (Rahman, 2023), along with an outline for a simulation for its evaluation. We name this *transcendental model selection*: the capacity to evaluate agents (including oneself) based on the structure of their generative models, specifically their hierarchical depth. This is complemented by *transcendental inference:* the mechanism by which cultural-level priors constrain lower-level inference and action through a precision parameter (α).

This framework:

- Explains why language is tied to identity: linguistic markers signal cultural model depth, enabling rapid assessment of whether strangers share the normative framework necessary for cooperation.
- Formalizes the “fourfold vision” Blake intuited: individual (*s*^(1)^), dyadic (*s*^(2)^), anonymous/shibboleth (*s*^(3)^), and cultural (*s*^(*L*)^) levels of social engagement, integrated through hierarchical inference.
- Generates testable predictions about developmental trajectories, neural correlates, cross-cultural variations, and digital communication patterns.
- Provides a foundation for building artificial systems capable of the flexible, multi-scale reasoning that defines general intelligence.

## 2 Transcendental inference and selection

This section introduces a novel computational implementation of active inference, building on prior studies in complex modeling, including meta-awareness and linguistics (Sandved-Smith et al., 2021; Friston et al., 2020). This formalization helps to establish how a model originally developed to stabilize social interactions through social typing was structurally repurposed for symbolic expression and thought. Stereotyping, moral typing, archetyping, and symbolic insignia all represent forms of categorizing complex conspecifics and one's own self-model. Moral typing, in this view, represents a categorical shift between natural semiotics (levels contained within the traditional generative process) and symbolic cognition (where the generative process is internalized within the model).

### 2.1 Formalizing transcendental inference

Transcendental inference implies hierarchical generative models with *L* levels of abstraction. The deepest latent states generate precision of prior beliefs, encoded by α, about subordinate latent states (*s*), based on observations (*o*). The aim is to minimize surprise, specifically, free energy *F*.

Free energy under such models decomposes into accuracy and increasingly high orders of complexity, contextualized by precision:


$$\begin{array}{l} F=\underset{\text{Accuracy}}{\underset{︸}{-E_{Q}\left[\ln\;P\left(o|s,m,\alpha \right)\right]}}+\underset{\text{Complexity}}{\underset{︸}{D_{KL}\left[Q\left(s,\alpha \right)||P\left(s,\alpha |o,m\right)\right]}} \end{array}$$ (1)

This subdivides the imperative of every agent to balance the trade-off between minimizing uncertainty in a parsimonious way, constructing models that are sufficiently accurate (benefit) without being overly complex (costly). This can be further decomposed to expose the hierarchical structure:


$$\begin{array}{l} F=E_{Q}\left[\ln\;Q\left(s,\alpha \right)-\ln\;P\left(o,s,\alpha |m\right)\right] \end{array}$$ (2)

$$\begin{array}{l} =\underset{\text{Accuracy}}{\underset{︸}{E_{Q}\left[-\ln\;P\left(o|s,\alpha ,m\right)\right]}}+\underset{\text{Complexity}}{\underset{︸}{D_{KL}\left[Q\left(s,\alpha \right)||P\left(s,\alpha |o,m\right)\right]}} \end{array}$$ (3)

$$\begin{array}{l} =\underset{\text{Accuracy}}{\underset{︸}{E_{Q}\left[-\ln\;P\left(o|s^{\left(1\right)},\alpha ,m\right)\right]}}+\underset{\text{Complexity: first-order}}{\underset{︸}{E_{Q\left(o\right)}D_{KL}\left[Q\left(s^{\left(1\right)}\right)||P\left(s^{\left(1\right)}|s^{\left(2\right)},\alpha ,m\right)\right]}} \end{array}$$ (4)

$$\begin{array}{l} +\underset{\text{Complexity: higher order}}{\underset{︸}{E_{Q\left(o\right)}D_{KL}\left[Q\left(s^{\left(2\right)}\right)||P\left(s^{\left(2\right)}|s^{\left(3\right)},\alpha ,m\right)\right]+\ldots +E_{Q\left(o\right)}D_{KL}\left[Q\left(s^{\left(L\right)}\right)||P\left(s^{\left(L\right)}|m\right)\right]}} \end{array}$$ (5)

$$\begin{array}{l} +\underset{\text{Transcendental}}{\underset{︸}{E_{Q\left(o\right)}D_{KL}\left[Q\left(\alpha \right)||P\left(\alpha |s^{\left(L\right)},m\right)\right]}} \end{array}$$ (6)

In simple terms, this equation shows how people can impulsively tune their behavior in alignment with norms and moral imperatives. We can flexibly navigate between our roles in numerous social groups, yet while attempting to optimize outcomes for a specific context or group, we can also avoid taking specific actions that might jeopardize our higher-order identity, i.e., one that aligns with a cultural type. Regardless of the benefits to my company, “I would simply not lie under oath,” as an example.

#### 2.1.1 Distinguishing transcendental inference from transcendental model selection

We distinguish two related but distinct concepts that together constitute the computational architecture of cultural cognition.

**Transcendental inference** is the mechanism by which cultural-level priors constrain lower-level inference and action. It refers to the hierarchical generative model structure (Equations 1–6 above) wherein the precision parameter (α) governs how strongly beliefs at the cultural level (*s*^(*L*)^) modulate expectations and actions at subordinate levels: individual (*s*^(1)^), dyadic (*s*^(2)^), and anonymous/shibboleth (*s*^(3)^). Transcendental inference explains how cultural norms influence individual behavior across various social contextual scales that involve potential competing demands on free energy minimization. When α is high, cultural-level beliefs dominate; an agent's actions remain constrained by broader normative expectations even when local or personal incentives would suggest deviation. When α is low, cultural constraints exert a weaker influence, and behavior responds more directly to immediate circumstances.

**Transcendental model selection** is the capacity to evaluate agents, including oneself, based on the *structure* of their generative models, specifically their hierarchical depth. It refers to the model comparison mechanism (Equations 7–9 below) that distinguishes between cultural models *m*(*s*^(*L*)^) and egocentric models *m*(*s*^(1)^). Transcendental model selection answers the more fundamental challenge when confronting anonymous conspecifics, which is a regular feature in human experience: how do we detect whether another agent operates at cultural depth or at a shallower, more egocentric level? This we label moral agency: the ability to type others as faithful to cultural norms (operating with deep hierarchical models that include cultural-level priors) and thereby the shared superordinate cultural identity, or as deviants (operating from narrower self-interest with shallow models that lack cultural depth).

Transcendental inference enables cultural coordination, while TMS enables the enforcement mechanisms that stabilize cultural coordination over time. When Pete declares Adam's violation (Section 3), Pete is engaging in transcendental model selection: inferring from Adam's behavior that Adam operates with a shallower model that prioritizes individual gain (*s*^(1)^) over collective norms (*s*^(*L*)^). Pete's declaration then activates transcendental inference in observers, raising α such that cultural-level expectations dominate their response to Adam.

This dual mechanism explains why moral violations feel categorically different from mere strategic failures. A hunter who fails to catch game has not revealed anything about model depth; misfortune is compatible with cultural faithfulness. A hunter who conceals food has revealed that his model lacks, or has deactivated, the cultural level that would constrain such behavior. The violation is not merely a bad outcome but evidence about model structure, triggering the distinctive phenomenology of moral judgment.

Technically, each order evaluates preferences and uncertainty minimization with respect to higher-order levels. The *s*^(*n*)^ variables correspond to increasingly complex levels of social organization, which carry over to general symbolic processing:

- *s*^(1)^ represents individual behavior
- *s*^(2)^ corresponds to social or dyadic expectations
- *s*^(3)^ reflects expectations based on shibboleth or tag-based social signifiers
- *s*^(*L*)^ (or *s*^(4)^ in our example) introduces a cultural level that abstracts expectations across various levels of social interactions, integrating them into a single cultural model

In the MOH, “objectivity” and “transcendence” are related but distinct concepts. Objectivity refers to the belief that moral principles exist independent of individual subjective perspectives—a “view from nowhere” that applies equally to all members of a cultural group (Nagel, 1989). Transcendence, meanwhile, refers to the cognitive mechanism that implements this belief: the capacity to adopt a perspective that extends beyond individual or dyadic models.

Computationally, objectivity manifests as shared prior beliefs at the cultural level *s*^*L*^, while transcendence is implemented through the precision parameter α that enables these cultural priors to constrain lower-level inferences. This distinction helps explain why moral beliefs feel both universal (objective) and culturally specific (transcendent)— they represent a particular cultural implementation of a general cognitive mechanism for perspective-taking beyond individual experience.

### 2.2 Model selection as moral agency

What brought us to the level of epistemic depth represented above? Moral agency. We are driven to detect “who is like me?” or “who we can trust?” From a first principles perspective, this simply amounts to whether a given agent is an in-group agent with moral agency, meaning do they have a hierarchical model that is deep enough to include the anonymous out-group.

Technically, transcendental inference is conditioned upon a particular generative model *m*, which can be optimized via Bayesian model selection or structure learning. This selection is based upon the differences in variational and expected free energy under the current model and an alternative model:


$$\begin{array}{l} \ln\;\frac{P\left(m|o\right)}{P\left(m^{\prime }|o\right)}=\ln\;\frac{P\left(o|m\right)}{P\left(o|m^{\prime }\right)}+\ln\;\frac{P\left(m\right)}{P\left(m^{\prime }\right)}=\Delta F+\Delta G \end{array}$$ (7)

$$\begin{array}{l} \Delta F=\ln\;P\left(o|m\right)-\ln\;P\left(o|m^{\prime }\right) \end{array}$$ (8)

$$\begin{array}{l} \Delta G=\ln\;P\left(m\right)-\ln\;P\left(m^{\prime }\right)=G\left(m\right)-G\left(m^{\prime }\right) \end{array}$$ (9)

At the most basic or foundational level, we evaluate agents by how “deep” they are. The numerator is the cultural model and the denominator the narrower version, representing the energetic response that occurs when witnessing a norm violation, i.e., what drives gossip when we learn about infidelity and physical intervention when we spot a mugger.

Technically, differences in variational free energy can be interpreted as a marginal prediction error between the two models in question. This equips transcendental inference with a further level of updating in terms of the model itself. Moreover, through the implementation of structured learning, agents are driven to infer fundamental factors or components of an agent's model. Combining transcendental inference and model selection (i.e., transcendent model selection), we can see how the generative process itself was endogenized, wherein agents are modeled off their own internal beliefs, leading to the virtual, cultural space as distinct from traditional agent-environment modeling.

In our framework, we designate:

- *m*:*m*(*s*^(*L*)^) as the cultural model
- *m*′:*m*(*s*^(1)^) as the egocentric model

Belief updating and free energy minimization (both variational and expected) generally account for how models change and how an agent selects between models. What does this form of model selection achieve? It offers a more insular evaluation of model structure as opposed to overall effectiveness, including orders of complexity (*s*^(*L*)^) in transcendental inference along with the factors implicit in models *m* at each level. This provides a rapid but deep view into an agent's priorities, individual/social trade-offs, future planning capacities, and more.

Functionally, this suggests an allowance for model comparison along several dimensions:

1. **Level of complexity** (hierarchical epistemic depth) as indicated by *s*^(*n*)^, quantifying the level of abstraction
2. **Activation** (versus inactivation) of a given level of hierarchy
3. **Salient factors** within a given level

While this comparison is limited to models of a certain type (i.e., sufficiently abstract), it may serve as the basis of our ability to traverse conceptual domains through metaphoric, analogical, or paradigmatic symbolic processing.

### 2.3 Convention and nature: the philosophical stakes of transcendental inference

The computational account above formalizes a distinction recognized in classical thought and a central theme of writers through the Renaissance, including Shakespeare (Cantor, 2013), (between *physis* (nature) and *nomos* (convention). Greek philosophers uniquely discovered that the political order is distinct from the natural world. *Physis* refers to things that grow and generate by themselves, independent of human will; *nomos* refers to things humans make or agree upon: laws, customs, money, and the belief that a particular person is king.

Transcendental inference implements the capacity to construct and inhabit conventional hierarchies. The cultural level (*s*^(*L*)^) is precisely the domain of *nomos*: symbolic structures and shared beliefs that exist because we collectively agree they exist. The “myth of objectivity” is the belief that these conventions carry the weight of natural reality and that moral principles exist independently of individual subjective perspectives. Critically, as grounded in Active Inference, it demonstrates that this consists merely of cognitive beliefs about beliefs.

However, such conventions are not opposed to nature but constitute the conditions under which human nature achieves its characteristic form. The “state of nature” for humans is not primitive wildness but civilization. To understand a thing, in the Aristotelian view, one looks at its highest development, not its lowest common denominator. An acorn's nature is the oak tree, not the seed. More broadly, Viktor Frankl and others argue that even finding one's place in a symbolic hierarchy is the very foundation of human wellbeing. Even under the adversity of a concentration camp, Frankl was able to find meaning and purpose as a source of resilience.

This philosophical framework illuminates what transcendental inference achieves beyond mere social coordination. Humans require *nomos* to realize their *physis*. Translated into the computational framework, transcendental inference creates the *virtual space* within which agents can develop capacities they could not develop in purely dyadic or individual contexts. Culture is not just the constraint imposed upon the individual; it is dynamically constructed within each agent's mind as a hierarchy. One that is not merely conventional (arbitrary); it is conventional-for-the-sake-of-natural-development. It is to be the “ideal” hunter, caretaker, etc. The precision parameter α governs how strongly this enabling structure shapes individual cognition and action.

Cultural scaffolding enables symbolic cognition. Language, planning, and abstract reasoning all require the adoption of perspectives that transcend individual or dyadic viewpoints. Morality, as the mechanism that originally instantiated and continues to maintain the cultural level, is thus constitutive of human cognitive capacity, not an external constraint upon it.

#### 2.3.1 The danger of collapsed convention

What happens when *nomos* collapses or when *physis* is reduced to mere animal appetite? This suggests a limitation and a warning. Transcendental model selection can detect whether agents operate with cultural depth. But the content of that cultural level matters. A demagogue's declarations can instantiate *false* cultural models, conventions that exploit rather than enable. The framework does not guarantee that cultural-level priors encode *good* values, only that they encode *shared* values that enable coordination and symbolic abstraction. The question of which cultural models enable human flourishing versus which corrupt it requires substantive moral philosophy, not merely computational mechanisms.

This maps to the α parameter in an important way. High α represents strong reliance on cultural-level priors: powerful for coordination but potentially rigid, capable of encoding prejudice as readily as wisdom. Low α represents responsiveness to direct experience: flexible but potentially unable to scale beyond intimate relationships or develop the symbolic capacities that require stable cultural scaffolding. The goal is not to maximize or minimize α but to achieve dynamic modulation: cultural priors that genuinely track human flourishing, combined with the capacity to recognize when those priors require revision.

## 3 Symbolic genesis: the deviant apple forager

To illustrate how moral typing created the virtual space for symbols, consider this stylized account (Wilson, 2015):

*Adam and Charles, foragers from different bands, meet at a common campsite. After exchanging vocalizations, i.e., primitive shibboleth, each band decides the others' sound sufficiently similar to trust*.

*Charles leads a foraging expedition; Adam joins. Returning successfully with berries, nuts, and apples, the group yields their harvest to elders, except Adam, who conceals an apple to court Eve from Charles's band*.

*Pete spots this deviation and declares it publicly. Charles, under the elders' gaze, must act. He confiscates the apple and banishes Adam and Eve, with tacit group acceptance*.

### 3.1 Ethnographic parallels

This stylized account captures dynamics documented across forager societies. The initial trust based on vocal similarity reflects tag-based cooperation observed in ethnographic research. The anthropologist Mervyn Meggitt recorded how Aboriginal groups from the Central and Western Desert articulate categorical distinctions through linguistic markers:

There are two kinds of blackfellows: we who are the Walbiri and those unfortunate people who are not. Our laws are the true laws; other blackfellows have inferior laws which they continuously break. Consequently, anything may be expected of these outsiders (Moffett, 2019a).

The Walbiri evaluate other groups by the quality of their cultural models, not a simple binary in/out group, nor whether they are deviant or aligned. The claim that others “continuously break their 'inferior laws” is an inference about model structure. It asserts that out-group members operate with deficient cultural-level priors (*s*^(*L*)^), rendering their behavior unpredictable from the Walbiri perspective. This is a transcendental model selection operating at the group level.

Pete's declaration and the subsequent group action illustrate what Christopher Boehm documents in his account of Cephu among the Mbuti Pygmies (Boehm, 2001). In that case, the tribe was involved in a group hunting expedition that required each hunter to form a semi-circle to trap the small game. Coordination thus enables group fitness. However, Cephu moved his hunting net ahead of others during the communal hunt, improving his personal catch at the collective's expense. Upon returning to camp,

“The other men relentlessly hurled insults at him. He was even denied a chair by a mere youth” (Wilson, 2015).

“Cephu attempted to cover his deviousness with a lie, but the band would hear nothing of it. They knew what had happened, and he knew that they knew what had happened”.

Two features of Boehm's account are significant here:

First, punishment extended beyond Cephu to his intimates: “The band seized it all, including a liver that his wife had hidden in anticipation of just such an eventuality”. This demonstrates that cultural enforcement operates through the dyadic level. Cephu's family is implicated not because they defected but because their identity is constituted through their association with him. Computationally, their Markov blanket overlaps with his; cultural-level consequences propagate through intimate-level connections. The *s*^(2)^ (dyadic) level is nested within the cultural enforcement mechanism.

Second, the group achieved consensus before acting. As Boehm explains, “this singularity of purpose is important because if a consensus is not built before action is taken, what might otherwise have been an instance of efficient group sanctioning can turn into sheer factional conflict, with both sides claiming moral rectitude”. This consensus-building is the social instantiation of transcendental model selection: the band collectively determines whether Cephu's behavior reveals deficient model depth before engaging enforcement. The declaration is performative, but it requires uptake; Pete's accusation becomes effective only when the group recognizes it as an accurate model assessment.

### 3.2 From moral typing to symbolic space

This narrative illustrates critical elements of symbolic genesis:

1. **Initial trust based on tag-based signaling (accent)**: The shibboleth level (*s*^(3)^) enables rapid categorization of strangers as potential cooperators based on linguistic markers, without requiring intimate history.
2. **Shared normative expectations**: The cultural level (*s*^(*L*)^) encodes expectations that apply uniformly across the anonymous in-group, creating predictability beyond dyadic relationships.
3. **Deviation detected and declared (moral typing)**: Transcendental model selection identifies Adam as operating with insufficient hierarchical depth, lacking or having deactivated the cultural-level constraints that would prevent his behavior.
4. **Cultural model reinforced through enforcement**: The punishment does not merely deter future violations; it instantiates the cultural level by demonstrating its causal power. The virtual space where cultural norms exist becomes real through their enforcement.

Pete's declaration constitutes a primitive speech act (Searle, 2010) that creates a moral type, simultaneously preserving the in-group model and establishing the virtual, cultural space where symbols acquire meaning beyond individual perspectives. The declaration works because it invokes a shared framework, the cultural level, that transcends Pete's individual perspective. In doing so, it demonstrates that such a shared framework exists and has consequences. This is the recursive foundation of symbolic cognition: symbols work because we believe they work, and moral enforcement provides the mechanism through which this belief becomes self-fulfilling.

## 4 Establishing a symbolic, cultural, or cosmic self

Declaring a moral belief reveals fundamental features of identity and permits a shared cultural Self. How can we understand identity from first principles and a scientific perspective? Michael Levin and Chris Fields' work, as they argue for a general view of semantic meaning in biology (Fields and Levin, 2020). Levin elsewhere defines identity and the Self in terms of what is meaningful or valued by an organism and its ability to computationally carry out these meaningfully valued goals (Levin, 2019). This charts the trajectory of both evolutionary (phylogenetic) and developmental (ontogenetic) forms of cohesive organization as a common value system. This can be seen, in their view, in basic biological functioning of stigmergy, i.e., a form of indirect coordination. Sharing of values and goals scales to more complex affective and emotional states, in particular, what is broadly described as the valence dimension of affect, the detection of good versus bad. This is a core feature of what defines our (conscious) identity (Hesp et al., 2021).

In the view of MOH, therefore, morality and declaration of certain core beliefs can singularly define identity and encourage others to adopt similar beliefs either consciously or unconsciously. Yet this is also adjacent to other forms of identification we still uniquely engage in. Language itself offers a form of identification separate from explicit belief.

### 4.1 Language and identity

Several implementations of active inference suggest that language requires an epistemic depth of generative models shared between speakers and interpreters (Friston et al., 2020; Sandved-Smith et al., 2021; Friston et al., 2024). These shared models require at least a tacit level of cooperation through shared perspectives on semantics, ground rules, or common reference frames (Vasil et al., 2020), even in adversarial communication paradigms. Consequently, language requires sharing of beliefs among agents (Friston et al., 2024). What Friston and colleagues term “federated inference” addresses the inherent question of how speakers/interpreters arrive at shared meaning:

[T]he meaning of a word just is (isomorphic to) the beliefs about the current state of the world and the narratives inherent in state transitions. [...] The emergence of shared meaning (cf., common ground) then reduces to an alignment of the (likelihood) mapping from belief states to words or symbols, among correspondents. (Friston et al., 2024)

However, shared meaning does not address the connection evident between language and identity. Previous accounts of language within active inference have ignored decades of research in the field of sociolinguistics and accent bias to claim that language can be understood simply as pragmatic action orientation (Tison and Poirier, 2021). This deeper layer of meaning was introduced in other areas of active inference literature, including a study on meta-awareness (Sandved-Smith et al., 2021), where it was presented as an internal view of thoughts and other mental actions. This layer formed first as a shared cultural identity prior to its inward focus in areas such as meditation, i.e., praying to supernatural entities, which preceded individual mindfulness meditation.

From the time infants are *in utero*, they prefer the sound of their mother's voice. From 6 months old, researchers have detected preferences for familiar accents (Kinzler, 2021), a bias with evolutionary and developmental roots that persists in adulthood and is observable at the level of sociology. From William Labov's studyin New York City through self-proclaimed third-wave sociolinguists, including Penelope Eckert, linguistic variation appears to reliably map to macrosociological and socioeconomic groups, as do individual positions within these perceived groups (Bell et al., 2016; Eckert, 2012).

Code-switching, as famously studied in the late Ruth Bader Ginsburg (Shapp et al., 2014), the practice of adjusting behavior or physical appearance to fit in with a specific context or group, is heavily geared toward the use of language:

Preliminary empirical review revealed that code-switching was a strategic tool for navigating and negotiating identities. It highlighted the practice's role in maintaining cultural heritage, aiding social integration, and enhancing professional advancement (Kipchoge, 2024).

The late Supreme Court justice varied her language to achieve her professional goals throughout her career, shielding her working-class accent as she entered high society, then finally dropped the status game once at the pinnacle of her career.

### 4.2 Transcendent claims of MOH

According to the MOH, the narrative layer that serves as a common reference frame for establishing shared semantics requires an additional, shared perspective, one that historically and developmentally involves a shift from dyadic to a triadic perspective. The MOH argues that this shift in cognitive capacity evolved hand-in-hand with morality, specifically our impulse to codify norms *universally* across those we designate as *human* within our anonymized in-group or tribe (Lorenz, 1974).

From an MOH perspective, the common ground formed among speakers, whether passively listening to someone on YouTube or shouting out our car window to a reckless driver or conversing with someone with whom we share a long history, is all subordinate to the belief in a higher common cultural or transcendent level of perspective. This transcendent perspective underwrites a common model of a shared reality (Laukkonen et al., 2025).

This aligns with Jacques Derrida's concept of transcendental signification, where he saw cultures as adopting hierarchical moral structures with particular deontic principles serving as grounding elements (Derrida and Bass, 1998). The MOH thus offers motivational stakes for the initial movement toward a broadly shared superordinate prior that grounds communicative belief sharing among cultural compatriots. Think of discourse in the Western world that references Hitler and the Holocaust as a common frame of reference that, for a time at least, motivated international cooperation on trade, common defense treaties, and international bodies like the UN and the World Bank.

To establish the above framework using alpha (α), we borrow from the computational views of meta-awareness (Sandved-Smith et al., 2021). The ability for meta-awareness and cognitive self-control, we argue, originated in the perception of an external hierarchical/cultural layer that serves as the precision parameter determining how strongly higher-level (cultural) beliefs constrain lower-level inferences. This relies on our ability to engage in transcendental inference, adopting a level deeper than dyadic or personal beliefs, allowing us to select between distinct hierarchical depths. This leveling up/down between cultural and individual levels of coordination is conditioned on a type of model selection.

I emphasize three important functions that this view of morality serves:

1. **Signaling** of allegiance to or deviation from cultural norms (e.g., gossip, finger-wagging)
2. **Precision-driven activation** of various inhabitable levels within the cultural hierarchy
3. **Initial implementation** of a superordinate cultural level through transcendental inference and structural learning (i.e., model selection)

### 4.3 Scaling opportunity/challenge

This confronts a core autopoietic assertion yet a computationally challenging problem of active inference: traversing scales of levels across distinct Markov blankets (Friston et al., 2025). One area where this occurs is planning as inference, or minimizing expected uncertainty over time. Model selection, i.e., structural learning framed here as moral agency, may offer an explicit connection between variational free energy (uncertainty minimization based on current observations) and the minimization of expected free energy (how policies or sets of actions will minimize uncertainty over future observations). A second challenge is understanding how individual agents minimize both their own and group uncertainty in ways that are distinct yet sufficiently aligned to constitute a joint blanket.

Transcendental inference and model selection offer the framework for a solution through the selection between a narrower model (*m*^*ego*^) and a broader one (*m*^*culture*^) that allows inference to extend into greater timescales, thereby underwriting policy selection and counterfactual planning. This model selection specifically motivates the information gain component of expected free energy (Parr et al., 2022). Another approach to scaling agents mirrors the uncertainty a given agent faces between levels. Building on recent studydemonstrating how agents scale their individual generative models to group-level models (using the Multi-Armed Bandit task), we suggest an extension of this model selection framework to archetypes, shifting from universal moral typing to one that permits culturally aligned subgroups defined along distinguishing features.

### 4.4 Human anonymity: from pleistocene to digital modernity

Transcendental inference deals with a recurrent theme in human civilization. The degree to which we engage with anonymous social agents has been tied to the so-called analytic vs. gestalt dichotomy, where cognitive focus of societies supposedly shifts variously between our tendency to parse concepts into lower syntax and our focus on the broad concepts themselves (Henrich, 2020; McGilchrist, 2019). Specifically a tendency toward anonymity, one that reigned during our early days as a species (Moffett, 2019b; Cohen, 2012) tends toward an analytic orientation, agrarian societies based on farming are gestalt, and finally western societies represent a return toward the analytic with heightened engagement with anonymity following institutional changes led by the catholic church but culminating in Lutheranism returned society to active engagement with increased anonymity.

Recent research (Albarracin et al., 2022) demonstrates how epistemic communities emerge across social networking platforms like X, where they display a tendency to reduce others to combinations of narrow prior expectations and their expressed beliefs on given topics (hashtags). This information-rich, perhaps ironically named “social networks,” display a hallmark of human experience. Our ability to entitize or abstract others based on our perception of their prior or expressed moral stance, in a way that reduces us to our most recently evolved parts of our biology.

We first inhabited intimate bands connected via shibboleth and eventual symbolic cultural insignia, then moved to sedentary agrarian societies that contained smaller urban regions that allowed anonymous interactions for things like trade and political organization. Industrialization subsequently expanded these smaller and denser regions, drawing more people from intimate rural areas, a trend that generally continues globally today. This progression toward greater anonymity is reaching a climax in the modern digital world, a dynamic captured in transcendental inference through the types of social scales we are able to inhabit and traverse.

## 5 Simulations and computational validation

### 5.1 Proposed simulation framework

To operationalize the transcendental model selection, we are actively developing a multi-agent simulation using the PyMDP framework. This simulation establishes conditions under which signaling-guided cooperation and coordination may emerge. Agents possess the capacity to gain from cooperation through cooperative scarce resource gathering, the ability to attend to distinct entities and direct both actions and signals toward them, and finally, the capacity to form higher-level beliefs about observed patterns that amount to typing others. We test whether signaling allows for the formation and learning of distinct communication, typing patterns, and associative learning, toward higher-order beliefs readable as impersonal social conventions, and ultimately whether the structure of the model itself can update to accommodate categorical inference about other agents' hierarchical depth.

We employ agents as hierarchical partially observable Markov decision processes (POMDPs) frequently leveraged in multi-agent reinforcement learning and active inference simulations, a cognitively-informed advance beyond traditional agent-based modeling in computational social science (Albarracin et al., 2022). The agents are uncertainty-minimizing, neurobiologically plausible generative models capable of perception, action, and learning. They interact with other agents and the world by observing and integrating multimodal sensory data, inferring beliefs and patterns of underlying hidden states, and selecting actions that minimize expected free energy. This framework enables the construction of hierarchical agents where lower levels form beliefs and pass them upward as observations to higher levels, which form beliefs about those beliefs and cascade updated predictions downward.

Critical to executing the POMDP is specifying vectors and matrices that define an agent's probabilistic beliefs about how observations, hidden states, and actions relate to one another. The *A* matrix (likelihood) informs perception by specifying how sensory observations (e.g., observing other agents' actions and signals, interoceptive hunger or arousal signals) relate to hidden states about oneself, others, and the world. The *B* matrix (state transitions) informs action by specifying how hidden states evolve over time given actions. The *C* matrix (preferences) specifies which observations the agent prefers, akin to rewards to be sought or “self-evidenced” in line with agents' desires and homeostasis. The *D* matrix (prior) specifies initial beliefs about hidden states, and the *E* matrix (habits) specifies beliefs about actions learned and relied upon when confronted with novel experiences. Each of these parameters regarding probabilistic beliefs can themselves be learned over time through further experience and exploration, as the Active Inference formulation further includes both reward-seeking (pragmatic value) and novelty-seeking exploration (epistemic value) as drivers of agent behavior (Parr et al., 2022; Friston et al., 2015).

### 5.2 Mapping hierarchical levels to agent versions

The simulation implements three agent versions that approximate the hierarchical levels described above:

**Version 1 (V1) agents** approximate the ego level (*m*^(1)^). These agents possess individual preferences regarding food, hunger, and valence, and they can attend to and model specific other agents through direct observation and experience. Signals such as smiling or frowning function as relatively direct indications of hidden states like emotional valence. V1 agents can learn about specific individuals through repeated interaction, but lack the capacity to sort agents into types. This approximates Pan-like social cognition, where coordination depends on familiarity and direct dyadic history.

**Version 2 (V2) agents** approximate the shibboleth level (*m*^(2)^) by incorporating an explicit in-group/out-group hidden state factor. Agents can now categorize others based on patterns and associations of observed actions, signals, and experiences rather than relying solely on intimate history. This is enabled by specifying in-group and out-group hidden states in the agent's higher-order model at *s*^(2)^, which are associated with various lower-order beliefs about intimate or dyadic interactions. Crucially, shibboleth-level coordination is not symbolic and is not unique to humans. Tag-based cooperation of this kind appears in other species, including naked mole rats, who use colony-specific odors to distinguish in-group from out-group members (Moffett, 2019a).

**Version 3 (V3) agents** approximate emergent typing through structure learning. We allow for structure learning rules to essentially self-specify the higher-order *m*^(2)^, where the structure of the typing itself is learned and modified purely through uncertainty minimization. This allows for testing if agents emergently find in-group and out-group typing to be most beneficial, or if some other taxonomy emerges. When scaled further with more agents, we can test for emergent forms of community formations, polarization, archetypal role-taking, and communicative or emotional signaling.

The transition from V2 to V3 represents the shift from pre-cultural to cultural cognition. V2 agents possess fragile models of strangers: sufficient for typing-based coordination under normal circumstances but narrow when violations occur. Because the shibboleth-level model compresses or caricatures the richness of intimate history, violations cannot be contextualized. The response amplifies agents' expectations based on in-group and out-group beliefs. Observers who can infer this structural feature have crossed into moral typing: they are modeling models, not merely behavior.

### 5.3 The alpha parameter

The cultural precision parameter (α) modulates how strongly higher-order beliefs influence lower-level inference and action selection. This parameter becomes operative at V2, where categorical higher-order typing beliefs exist to exert such influence. α here acts like a “gain” or amplification term, modulating the influence of higher-order typing expectations on lower-order beliefs and decision-making. When α is high, higher-order typing beliefs strongly influence and bias perception and decision-making. If Charles determines that Adam belongs to the in-group through their interactions, a high α means Charles will expect Adam to display in-group characteristics and will weigh those expectations heavily relative to direct observations. Deviations from expected behavior will then generate substantial surprise. When α is low, however, Charles learns and updates beliefs more directly from experience with Adam, specifically at the lower level *m*^(1)^, with less (if any) influence from Charles' higher-order typing beliefs.

We envision an important function of α as a cultural conformity mechanism. High α emphasizes higher-order categorical priors, the kind of cognition that enables rapid coordination with strangers but also produces rigidity and stereotyping. Low α, conversely, may represent cognition more responsive to direct experience, flexible but potentially unable to scale beyond intimate relationships.

### 5.4 Testable predictions

The simulation enables several empirical tests:

1. **Signal migration**: We can observe whether signals that begin as direct indications of valence come to carry information about group membership, expected behaviors, and cooperativeness, and eventually about model structure. Do agents converge on shared signal meanings? Does signal reliability (the correlation between signal and subsequent behavior) differ across agent versions?
2. **Fitness comparison across versions**: Do V2 agents outperform V1 agents when stranger encounter rates are high? Do V3 agents outperform V2 agents when norm violations occur? The prediction is that hierarchical depth provides advantages that scale with social complexity and uncertainty. What costs come about as stereotyping emerges even in this simplified atavistic model? How do the trade-offs shift as environmental dynamics are more/less scarce or regenerative?
3. **Structure learning conditions**: Do V3 agents spontaneously develop categorical representations resembling moral types? What conditions trigger this development? The prediction is that structure learning occurs specifically when fragile (shibboleth-level) models encounter violations they cannot contextualize.
4. **α** **parameter effects**: We can vary α parametrically and observe effects on coordination, flexibility, and fitness. The prediction is that intermediate α values optimize the tradeoff between cultural coordination and responsiveness to direct experience, with optimal values depending on environmental stability and the rate of stranger encounters.

### 5.5 Validation through existing simulations

Two recent simulations inspire a similar application to this computational account of MOH. First is Shreesha and colleagues (Shreesha et al., 2025), who apply game theory to the emergence of individual cells to multicellular structures. They specifically use a modified version of the prisoners' dilemma, relaxing the assumption that the number and boundaries of agents are fixed, building on theoretical and other simulations from Michael Levin's prior study (Levin, 2019).

Second is Thestrup Waade et al. (2025), who demonstrate scaling from individual to emergent group-level generative models. Their Multi-Armed Bandit framework shows how individual agents can form coherent group-level inference patterns.

Building on these foundations, we propose extending their frameworks to test how societies move beyond flat moral distributions, where all are bound by core codified norms [in the spirit of Boehm (2001, 2012)'s study on egalitarian hunter-gatherer societies], toward nuanced archetypes that follow a hierarchy of nested types.

### 5.6 Proposed archetype extension

We propose a staged simulation architecture that tests whether hierarchical cognitive depth enables archetypal specialization. Rather than mapping archetypes directly onto existing agent node types, we implement three agent versions that progressively add representational layers. Version 1 (V1) agents operate with a single level of inference: each agent maintains mental representations of every other agent it encounters (e.g., Adam maintains beliefs about [“Adam”, “Charles”, “Eve”]) and coordinates through direct dyadic learning. V1 approximates the intimate scale of social cognition, where cooperation depends on familiarity rather than categorical abstraction.

Version 2 (V2) agents augment this base with an explicit higher-order level. In addition to their level 1 representations, V2 agents possess hard-coded categorical schemas, such as [“Sage”, “Citizen”, “Soldier”] or [“In-group”, “Out-group”], through which they classify and predict the behavior of other agents. Level 2 beliefs receive observations from level 1 (updated beliefs about attention, valence, and resource states) and in turn modulate lower-level inference through precision weighting. These categorical schemas enable sparser, faster coordination with less-familiar agents but at the cost of contextual nuance. Though archetypes like sages and soldiers may not seem like obligatory roles alongside the daily demands of foraging and childcare, they are worth distinguishing for their orientation toward collective goods: soldiers and sages seek security and knowledge, respectively, not ostensibly for individual betterment but for the group. In economic terms, these constitute public goods whose contribution can be more readily measured against the cultural entity.

Version 3 (V3) agents replace the hard-coded level 2 with structure learning, where higher-order representations are not pre-specified but discovered through experience. When an agent's existing model fails to reduce free energy despite continued interaction, structural reorganization may be triggered, adding new representational capacity. V3 agents may converge on categorical distinctions resembling in-group/out-group or archetypal roles, or they may develop taxonomies we have not anticipated.

This staged design yields testable predictions across agent versions:

- Whether V2 agents, equipped with archetypal categories, coordinate more efficiently than V1 agents as stranger encounter rates increase.
- Whether V3 agents spontaneously develop specialized categories resembling archetypes when environmental and social complexity demand them.
- Whether group-level fitness depends on a balanced distribution of categorical types, and whether such balance emerges naturally or requires exogenous specification.

### 5.7 Digital platforms as natural experiments

We can extend recent studies dealing with epistemic beliefs in Twitter. Albarracin et al. (2022) demonstrate how expressing binary beliefs creates epistemic communities through confirmation bias using hashtags on social networks. Although Twitter was not used by our ancestors, modern digital platforms offer a space where ancient and primal tendencies of moral type reduction not only emerge but are sharpened due to a critical and often underrated dimension of human distinction: anonymity.

In Moffett (2019b)'s account, this is the purpose of our impulse to stereotype, to identify with anonymous members. Normally, of course, this inherent act of sparse agent reduction is attenuated through dyadic interactions. Charles and Peter had to contend with those who may have witnessed Peter's earlier attempts to court Eve, thus weakening his signal attempt.

Albarracin et al.'s model can be formalized within Transcendental Inference. Moral signaling and confirmation bias interact to create echo chambers, particularly in environments without the “ower order” (i.e., social/intimate) buffers. X represents an environment of runaway “Peters” able to express beliefs about topics, including other people's types, experiencing no enforcement from Charles or other intimates.

Their simulations demonstrated that when agents preferentially sample information from like-minded others (high epistemic confirmation bias γ), polarized epistemic communities naturally emerge:


$$\begin{array}{l} \langle \rho \rangle =\frac{1}{S}\overset{S}{\underset{s=1}{\sum }}\rho_{s} \end{array}$$ (10)

Where ρ_*s*_ is the polarization index for a specific simulation trial, measuring the spread of beliefs across agents, and 〈ρ〉 is the average polarization across many simulations.

Crucially, their findings showed that polarization emerges through a complex interaction between:

1. **Network connectivity** (*p*): Sparser networks (lower *p*) increase polarization.
2. **Epistemic confirmation bias** (γ): Higher bias increases polarization.
3. **Social volatility** (ω_*Soc*_): Beliefs about how quickly others' opinions change.
4. **Habit formation** (η): The tendency to repeatedly sample the same sources.

This framework helps explain why moral declarations are so powerful in human groups; they leverage these mechanisms to reinforce group boundaries and belief structures. The MOH suggests that these mechanisms evolved precisely because they facilitated the formation of stable, coherent cultural identities through moral signaling.

Their framework can be adapted to test specific predictions of this moral archetype hypothesis:

1. **Parameter mapping**: The α parameter can be reinterpreted as moral conformity pressure rather than decisiveness.
2. **Proposed simulation**: Modify to include multiple norm domains (cooperation, fairness, harm) rather than just win/loss outcomes, with variable cultural pressure across different contexts.
3. **Testable predictions**: Whether specialized archetypes emerge naturally, improve group-level inference, and require balanced distribution for cultural persistence.

## 6 Addressing AGI safety concerns

### 6.1 Positioning TMS in the AGI landscape

The contemporary AGI conversation clusters around several schools: (1) active inference and hierarchical generative models; (2) alignment and safety through value learning; (3) embodied and cultural cognition; (4) philosophical foundations of mind and semiotics; and (5) practical skepticism about imminence. Transcendental model selection engages meaningfully with all five but occupies a distinctive position that requires articulation.

Here, we propose that morality and moral agency constitute the mechanism by which cultural constraints integrate across multiple scales of social interaction. This differs from claiming that culture shapes values (a commonplace in embodied cognition) and from claiming that agents must be aligned with pre-specified values (the alignment literature). Instead, TMS argues that the very capacity for symbolic thought emerges from, and requires, the ability to make meta-level model selections based on moral typology. This is stronger than value learning and more mechanistic than philosophical phenomenology.

Most AGI frameworks treat culture as background context or as a learning problem separate from the core aim to build intelligence. TMS treats culture and morality as generative mechanisms with computational substance, specifically the precision parameter α that weights cultural models over egocentric ones and the model selection mechanism (Equations 7–9) that evaluates hierarchical depth.

### 6.2 Technical cognition and the question of replication

Katherine Hayles distinguishes three forms of cognition: (1) human cognition (conscious, symbolic, reflective), (2) animal cognition (adaptive but largely non-symbolic), and (3) technical cognition (computational processes that process information without phenomenal experience) (Hayles, 2017). Her analysis suggests that technical cognition is genuinely cognitive but operates through different mechanisms than human cognition, raising the question of whether “general” intelligence as humans exhibit it can be replicated in systems lacking embodied social development.

Hayles decentralizes human intelligence and distributes cognition across biological and technological systems. TMS does not contradict this position but adds specificity: while cognition may be distributed across systems, some systems (humans, potentially AGI) achieve a kind of integration across scales that requires explicit model selection. TMS accepts Hayles's distributed cognition but argues that the capacity to integrate multiple scales through moral agency is architecturally distinct due to evolutionary history (foragers to sedentary to industrial to digital societies), not fundamental essence.

Human general intelligence, on the TMS account, emerged through a specific evolutionary pathway: morality created the cultural scaffolding that enabled symbolic abstraction, which in turn enabled the flexible, context-sensitive reasoning characteristic of human cognition. This pathway involved social pressure (group-against-group competition), embodied development (infants acquiring cultural models through extended caregiving), and recursive self-modeling (agents modeling agents modeling cultural norms).

### 6.3 Differentiating TMS from related frameworks

**Against standard active inference**: Friston (2010) treat hierarchy and precision as optimization mechanisms for reducing free energy across timescales. TMS argues that moral agency, the ability to evaluate and select between models at different epistemic depths, is the mechanism by which cultural levels become subordinating rather than merely hierarchical. It is not simply that deep models better predict; agents detect depth as a moral marker and adjust their own precision accordingly. This is structural learning with a specific functional signature. Model selection (Equation 7) is a novel addition to Friston's hierarchy, not a restatement of it.

**Against pure value alignment literature**: Bostrom, Yudkowsky, and others frame the problem as: “How do we ensure AGI optimizes for human values?” TMS reframes it: “How do we ensure AGI acquires human values the way humans do, through cultural embedding, moral practice, and the integration of multiple social scales?” This shifts from static value specification to dynamic, developmentally grounded learning. TMS does not solve the alignment problem but provides a framework for understanding how values could be implicitly learned through cultural embedding, which offers more traction than hand-wavy appeals to “human values.”

**Against embodied cognition alone**: Clark and Chalmers (1998) show how cognition depends on embodied action and environmental scaffolding. TMS adds that moral agency is the mechanism by which embodied agents transcend individual and dyadic scales into cultural coordination. Embodiment is necessary but not sufficient; explicit model selection over multiple social depths is required. Thompson's concept of autonomy as self-individuation, the capacity of a system to maintain itself in precarious conditions and thereby establish a normative perspective, provides phenomenological grounding: moral agency (model selection) can be interpreted as a way autonomous systems evaluate whether others maintain the same kind of self-maintaining perspective.

### 6.4 Implications for building AGI

I adopt a version of the replication view: AGI requires implementing hierarchical architecture including mechanisms for transcendental inference (cultural-level priors constraining lower-level processing) and transcendental model selection (evaluating model depth in self and others). Systems lacking these mechanisms may achieve narrow competence but will fail at the flexible, multi-scale reasoning that defines general intelligence.

However, the relevant architecture is not individual but collective. Human general intelligence is not located in single brains but emerges from the integration of multiple agents facing joint social and ecological challenges: fighting other groups, foraging, hunting, shared childcare. The information space these agents traverse motivates both rapid response (immediate problem-solving) and long-term modeling (cultural learning across generations).

For artificial systems, this suggests AGI may require not a single model but an ecology of models operating across each other, facing genuine challenges requiring both immediate adaptation and extended planning. The cultural level (*s*^(*L*)^) in such systems would emerge from coordination requirements of multi-agent interaction, not from top-down specification of values.

### 6.5 Value alignment reconceived

Traditional approaches struggle with value specification: how to formally define human values for optimization. Transcendental model selection sidesteps this by learning values implicitly through cultural model acquisition, similar to human development.

The system does not optimize a fixed utility function but dynamically switches between value systems based on context. This mirrors how humans balance self-interest with social responsibility rather than being purely altruistic or selfish. This can be used both within and across systems. The first offers a pathway for building AGI aligned with human perspectives, and the latter offers a pathway for building a regulatory or governance system that regulates private AGI systems.

The risk, as the physis/nomos analysis suggests, is that systems may learn biases embedded in their cultural training as readily as wisdom. Detecting and correcting such biases requires not programmatic intervention (which presumes we can specify correct values in advance) but ongoing moral discourse: the same recursive process through which human cultures revise their norms. AGI systems that participate in this discourse, rather than simply optimizing fixed objectives, may be more robustly aligned precisely because their values remain contestable. This sketch remains speculative but suggests concrete simulation targets for future studies (e.g., testing corrigibility as context-conditioned model switching in multi-agent POMDPs).

### 6.6 Corrigibility

The capacity for model selection provides natural corrigibility. When, say, a human oversight operator signals the need for intervention, the system can switch to models encoding deference and cooperation rather than pursuing its current objective single-mindedly. Alternatively, an MOH-enabled governance system can detect potential norm violations swiftly and hence advise, influence, or override third-party systems.

This emerges from the same mechanism humans use to recognize when to defer to authority or expertise: transcendental model selection identifies contexts where operating at individual depth (*s*^(1)^) is inappropriate, and transcendental inference raises α to give cultural-level constraints (including deference to oversight) priority.

### 6.7 Robustness

Multi-scale validation provides robustness against distribution shift and adversarial inputs. Actions must be coherent across multiple model depths: individual preferences, dyadic expectations, anonymous conventions, and cultural norms. This prevents both impulsive responses (egocentric only) and rigid rule-following (cultural only without context sensitivity).

The biological precedent suggests this architecture is robust to the kinds of challenges AGI will face in open-ended environments with competing objectives and stakeholders.

## 7 Limitations and scope

The transcendental model selection framework, while offering a novel computational account of cultural cognition and symbolic genesis, has important limitations that bound its current explanatory scope.

### 7.1 Coercion, inequality, and asymmetric power

The framework models coordination and norm enforcement among agents with roughly symmetric capacities for moral agency. However, much of human history involves asymmetric power relations: colonial impositions of cultural models, institutional coercion of minorities, and systematic exclusion of groups from moral consideration.

TMS can describe such situations formally. Colonial imposition represents one cultural model ($s_{colonizer}^{\left(L\right)}$) suppressing another ($s_{colonized}^{\left(L\right)}$), often by denying that the colonized possess cultural depth at all, a form of transcendental model selection that types entire populations as lacking moral agency. The framework can thus illuminate the *cognitive mechanisms* of domination, but it does not currently model the *material conditions* (military power, economic control) that enable such imposition.

Future extensions should integrate TMS with models of resource competition and coercive capacity. The Markov boundary between transcendental models might be conceptualized not as a permeable membrane enabling mutual influence but as a contested frontier where power determines whose model prevails.

### 7.2 Bias reinforcement

If systems (human or artificial) learn cultural models implicitly, they may learn the biases embedded in those cultures. The α parameter could amplify existing prejudices: high α means cultural-level priors dominate inference, and if those priors encode racism, sexism, or other discriminatory patterns, high α will produce discriminatory behavior.

Two considerations temper this concern. First, to the extent that humans remain morally agentic, capable of revising their moral commitments through reflection and discourse, we cannot and should not seek to programmatically eliminate bias. Legal institutions and private organizations can police and encourage prosocial behavior, but they cannot correct what lies within the human heart. The same applies to AI systems: if they are genuinely agentic, their values will be revisable through discourse rather than fixed by design.

Second, TMS can shed light on bias through rigorous analysis of how cultural-level priors influence lower-level inference. We can model, for instance, how persistent preferences for an in-group correlate with the devaluation of an out-group across generations, or how particular configurations of α amplify versus attenuate discriminatory patterns. This diagnostic capacity does not guarantee solutions but enables the kind of understanding that precedes intervention.

### 7.3 Subcultural conflict

The archetype extension (soldier, sage, citizen) assumes archetypes exist within a coherent cultural hierarchy. However, what about genuinely competing value systems? Religious conflicts, political polarization, and ideological warfare involve subcultures with incompatible *s*^(*L*)^ priors that cannot be reconciled by adjusting α.

The framework predicts that such conflicts will be characterized by mutual transcendental model selection: each side typing the other as lacking moral depth, operating from corrupted or shallow models. The Walbiri statement, “Our laws are the true laws; other blackfellows have inferior laws which they continuously break”, exemplifies this pattern. Each side views itself as operating at cultural depth and the other as deficient.

Resolution, on this account, requires either (a) establishing a higher-order shared framework that encompasses both subcultures, effectively creating a meta-cultural level *s*^(*L*+1)^, or (b) partitioning into separate cultural systems with managed boundaries. The framework does not predict which outcome will occur; that depends on material conditions, leadership, and contingent historical factors beyond the cognitive mechanisms TMS describes.

### 7.4 Deliberate manipulation

If moral declarations function as performative utterances that instantiate cultural levels, bad actors can exploit this mechanism. A demagogue's declarations could create false cultural models. Shared beliefs that serve the demagogue's interests rather than collective flourishing. The framework does not inherently distinguish genuine moral declarations from strategic manipulations; both work through the same mechanism.

This limitation is partially addressed by the consensus requirement that Boehm documents. Declarations become effective only when the group recognizes them as accurate assessments. Pete's accusation requires observers to independently recognize Adam's behavior as evidence of shallow model depth. A demagogue's manipulation succeeds without this verification, i.e., when audiences accept declarations without independent assessment.

The digital environment is particularly vulnerable because it removes the lower-order buffers (intimate knowledge, dyadic history) that normally attenuate crude moral typing. Online platforms create conditions where “runaway Peters” can make declarations without accountability, producing polarized epistemic communities through confirmation bias. The framework diagnoses this pathology but does not prescribe solutions beyond the general principle that robust cultural cognition requires functional intermediate levels (*s*^(2)^, *s*^(3)^) between individual and cultural scales.

### 7.5 What the framework cannot address

Some phenomena lie outside the current framework's scope:

- **Individual variation in moral development**: TMS describes the architecture shared across normally developing humans but does not model individual differences in moral capacity, psychopathy, or developmental disorders affecting social cognition.
- **Non-human moral cognition**: The framework is designed for human-style cultural cognition. Whether other species possess analogous mechanisms, and whether the framework can be extended to model their social cognition, remains unexplored.
- **Normative ethics**: TMS describes how cultural norms function computationally, not which norms are correct. It is compatible with moral realism, relativism, and various metaethical positions without adjudicating between them.
- **Historical contingency**: The framework models cognitive mechanisms but not the historical processes through which particular cultures developed particular norms.

These limitations do not invalidate the framework but define its scope. TMS offers a computational account of the cognitive architecture enabling cultural cognition. Integrating this account with models of power, history, and normative ethics remains work for future research.

## 8 Discussion

This study has presented a formal computational account of the Myth of Objectivity Hypothesis, demonstrating how morality and symbolic thought co-evolved within a transcendental inference mechanism. By formalizing the hierarchical structure that enables humans to integrate individual, dyadic, anonymous, and cultural levels of social interaction, we aimed to show how model selection between egocentric and cultural models enables the precision-weighted activation of different social scales. This framework explains the origins of symbolic communication as well as the persistent connection between language and identity observed across cultures from Johannesburg to New York City.

In the biblical account, Adam and Eve were given a flaming sword after they were cast out of paradise. The metaphor in this study, of course, is their newly found powers of symbolic expression. The narrative here illustrated how moral declarations function as performative utterances that instantiate cultural levels of inference, moving from tag-based cooperation within anonymous social units to create shared virtual spaces necessary for symbolic reasoning. Through transcendental inference, we believe our ancestors developed the capacity to adopt perspectives that transcend individual subjectivity while maintaining the flexibility to traverse multiple levels of social abstraction through model selection.

This computational framework generates testable predictions about developmental trajectories, neural correlates, cross-cultural variations, and digital communication patterns. The simulation outline details how we plan on doing this and how similar validations can be carried out. The extension to archetypal specialization (soldiers, sages, citizens) demonstrates how cultural-level models can differentiate while maintaining coherence, offering insights into both historical social organization and contemporary epistemic polarization in digital environments.

Perhaps most significantly, this study illuminates fundamental principles of intelligence itself. Active inference emphasizes that organisms minimize uncertainty hierarchically across multiple scales–from cellular to social to cultural levels. This hierarchical organization appears integral to any complex intelligent system. Humanity's distinctive achievement can be written down as transcendental inference: the ability to arbitrarily select between different hierarchical depths through model selection, enabling seamless navigation between intimate conversations, anonymous social interactions, and abstract symbolic reasoning within a unified cognitive architecture.

Any artificial general intelligence must similarly develop explicit mechanisms for traversing hierarchical scales, integrating multiple Markov blankets, and selecting appropriate levels of abstraction for different contexts. The precision parameter α that enables cultural-level constraints to guide individual behavior may be crucial for AGI systems that need to operate coherently across personal, social, and institutional scales. Without this capacity for transcendental model selection, artificial systems risk remaining trapped within single scales of analysis, fundamentally limiting their ability to achieve human-level general intelligence, and posing potential risks by not taking governance factors into account. In other words, this offers both a practical system of intelligence and one with an embedded alignment to deeper cultural values.

The Myth of Objectivity Hypothesis reveals that our symbolic capacity emerged from constructing shared beliefs in transcendent perspectives that enabled scale-free forms of social coordination. The myth of objectivity continues to underwrite our language, institutions, and moral systems. Understanding its computational foundations may be essential not only for explaining human uniqueness, but for building artificial minds capable of the flexible, multi-scale reasoning that defines general intelligence.

## Data Availability

The original contributions presented in the study are included in the article/Supplementary material, further inquiries can be directed to the corresponding author.
