# Supplementary material for: Transcendental model selection: a computational account of symbolic cognition and general intelligence through morality and culture
Source: Front Sociol. 2026 Apr 22;11:1646503. doi: 10.3389/fsoc.2026.1646503 (PMC13143734; doi:10.3389/fsoc.2026.1646503)
Supplement: Supplementary file 1 [file Supplementary_file_1.pdf]

Supplementary Material

1 SUPPLEMENTARY MATERIAL: KEY EQUATIONS FOR VARIATIONAL AND EXPECTED FREE ENERGY

Inference about hidden states (i.e., state estimation) corresponds to inverting a generative model, given a sequence of outcomes, while *learning* corresponds to updating model parameters. The requisite expectations constitute the sufficient statistics μ of posterior beliefs $Q(s, \pi, a|\mu)$ about states of affairs in the world s, actions u and model parameters a, respectively. This mean field factorization effectively partitions model inversion into inference, planning and learning.

1.1 Variational Free Energy and Inference

In variational Bayesian inference (a.k.a., approximate Bayesian inference), model inversion entails the minimization of variational free energy with respect to the sufficient statistics of approximate posterior. This can be expressed as follows, where, for clarity, we will deal with a single factor, such that the policy (i.e., combination of paths) becomes the path, π. Omitting dependencies on previous states, we have for model m:

$$F(o, \mu) = E_{Q(s|\mu)}[\ln Q(s|\mu) - \ln P(o, s|m)] \tag{S1}$$
$$= E_{Q(s|\mu)}[\ln Q(s|\mu) - \ln P(s|o, m)] - \ln P(o|m) \tag{S2}$$
$$= D_{KL}[Q(s|\mu)||P(s|o, m)] - \ln P(o|m) \tag{S3}$$

Because the (KL) divergences cannot be less than zero, the penultimate equality means that free energy is zero when the (approximate) posterior is the true posterior. At this point, the free energy becomes the negative log evidence for the generative model (Beal, 2003). This means minimizing free energy is equivalent to maximizing model evidence.

Planning emerges under active inference by placing priors over (controllable) paths to minimize expected free energy (Friston et al., 2015):

$$G(\pi) = E_{Q(o_\tau, s_\tau, a|\pi)}[\ln Q(s_\tau|\pi) - \ln Q(s_\tau|o_\tau, \pi) - \ln P(o_\tau|m)] \tag{S4}$$
$$= E_{Q(o_\tau, s_\tau, a|\pi)}[\ln Q(s_\tau|\pi) - \ln Q(s_\tau|o_\tau, \pi)] - E_{Q(o_\tau|\pi)}[\ln P(o_\tau)] \tag{S5}$$
$$= \underbrace{-E_{Q(o_\tau|\pi)}[D_{KL}[Q(s_\tau|o_\tau, \pi)||Q(s_\tau|\pi)]]}_{\text{epistemic value}} - \underbrace{E_{Q(o_\tau|\pi)}[\ln P(o_\tau)]}_{\text{extrinsic value}} \tag{S6}$$

Here, $Q(o_\tau, s_\tau, a|\pi)$ is the posterior predictive distribution over parameters, hidden states and outcomes at the next time step, under a particular path. Note that the expectation is over *observations in the future*, hence, *expected* free energy. This means that preferred outcomes—that subtend expected cost and risk—are prior beliefs, which constrain the implicit planning as inference (Attias, 2003; Botvinick and Toussaint, 2012; Van Dijk and Polani, 2013).

One can also express the prior over the parameters in terms of an expected free energy, where, marginalizing over paths:

$$P(a|m) \propto \exp(-\beta \cdot G(a)) \tag{S7}$$

$$G(a) = D_{KL}[Q(o,s|a)||P(o|c)] - E_{Q(o|a)}[H[Q(s|o,a)]] \tag{S8}$$

where $Q(o, s|a)$ is the joint distribution over outcomes and hidden states, encoded by Dirichlet parameters. Note that the Dirichlet parameters encode the mutual information, in the sense that they encode the joint distribution over outcomes and their hidden causes. Expected free energy can be regarded as a universal objective function that augments mutual information with expected costs or constraints. Constraints—parameterized by c—reflect the fact that we are dealing with open systems with characteristic outcomes, o. This can be read as a constrained principle of maximum mutual information or minimum redundancy (Ay et al., 2008; Barlow, 2012; Linsker, 1990; Olshausen and Field, 1996). In machine learning, this kind of objective function underwrites disentanglement (Higgins et al., 2021; Sanchez et al., 2019), and generally leads to sparse representations (Gros, 2009; Olshausen and Field, 1996; Sakthivadivel, 2022; Tipping, 2001).

There are many special cases of minimizing expected free energy. For example, maximizing expected information gain maximizes (expected) Bayesian surprise (Itti and Baldi, 2009), in accord with the principles of optimal experimental design (**?**). The implicit resolution of uncertainty is related to artificial curiosity (Schmidhuber, 1991; **?**) and speaks to the value of information (Howard, 1966). Expected complexity or risk is the same quantity minimised in risk sensitive or KL control (Klyubin et al., 2005; Broek et al., 2012), and underpins (free energy) formulations of bounded rationality based on complexity costs (Braun et al., 2011; Ortega and Braun, 2013) and related schemes in machine learning; e.g., Bayesian reinforcement learning (Ghavamzadeh et al., 2015). Finally, minimizing expected cost subsumes Bayesian decision theory (Berger, 1988).

REFERENCES

Attias, H. (2003). Planning by Probabilistic Inference. In *Proceedings of the Ninth International Workshop on Artificial Intelligence and Statistics*, eds. C. M. Bishop and B. J. Frey (PMLR), vol. R4 of *Proceedings of Machine Learning Research*, 9–16

KEY: attias$_{planning_2}$003
ANNOTATION: $ReissuedbyPMLRon01April2021.$

Ay, N., Bertschinger, N., Der, R., Güttler, F., and Olbrich, E. (2008). enPredictive information and explorative behavior of autonomous robots. *The European Physical Journal B* 63, 329–339. doi:10.1140/epjb/e2008-00175-0

Barlow, H. B. (2012). Possible Principles Underlying the Transformations of Sensory Messages. In *Sensory Communication*, ed. W. A. Rosenblith (The MIT Press). 216–234. doi:10.7551/mitpress/9780262518420.003.0013

Beal, M. (2003). *Variational algorithms for approximate Bayesian inference*

Berger, J. O. (1988). eng*Statistical decision theory and bayesian analysis*. Springer series in statistics (New York Berlin Heidelberg [etc.]: Springer), 2nd ed edn.

Botvinick, M. and Toussaint, M. (2012). enPlanning as inference. *Trends in Cognitive Sciences* 16, 485–488. doi:10.1016/j.tics.2012.08.006

Braun, D., Theodorou, Evangelos, Ortega, Pedro, and Schaal, Stefan (2011). Path Integral Control and Bounded Rationality

[Dataset] Broek, B. v. d., Wiegerinck, W., and Kappen, H. (2012). Risk Sensitive Path Integral Control. doi:10.48550/arXiv.1203.3523. ArXiv:1203.3523 [cs]

KEY: broek$_{r}isk_{2}012$

ANNOTATION: $Comment : AppearsinProceedingsoftheTwenty - SixthConferenceonUncertaintyinArtificialIntelligence(UAI2010)$

Friston, K., Rigoli, F., Ognibene, D., Mathys, C., Fitzgerald, T., and Pezzulo, G. (2015). enActive inference and epistemic value. *Cognitive Neuroscience* 6, 187–214. doi:10.1080/17588928.2015.1020053

Ghavamzadeh, M., Mannor, S., Pineau, J., and Tamar, A. (2015). Bayesian Reinforcement Learning: A Survey. *Foundations and Trends® in Machine Learning* 8, 359–483. doi:10.1561/2200000049. ArXiv:1609.04436 [cs]

[Dataset] Gros, C. (2009). Cognitive computation with autonomously active neural networks: an emerging field. doi:10.48550/arXiv.0901.3028. ArXiv:0901.3028 [q-bio]

KEY: gros$_{c}ognitive_{2}009$

ANNOTATION: $Comment : keynotereview.CognitiveComputation(inpress, 2009)$

Higgins, I., Chang, L., Langston, V., Hassabis, D., Summerfield, C., Tsao, D., et al. (2021). Unsupervised deep learning identifies semantic disentanglement in single inferotemporal neurons. *Nature Communications* 12, 6456. doi:10.1038/s41467-021-26751-5. ArXiv:2006.14304 [q-bio]

Howard, R. (1966). Information Value Theory. *IEEE Transactions on Systems Science and Cybernetics* 2, 22–26. doi:10.1109/TSSC.1966.300074

Itti, L. and Baldi, P. (2009). enBayesian surprise attracts human attention. *Vision Research* 49, 1295–1306. doi:10.1016/j.visres.2008.09.007

Klyubin, A., Polani, D., and Nehaniv, C. (2005). Empowerment: A Universal Agent-Centric Measure of Control. In *2005 IEEE Congress on Evolutionary Computation* (Edinburgh, Scotland, UK: IEEE), vol. 1, 128–135. doi:10.1109/CEC.2005.1554676

Linsker, R. (1990). enPerceptual Neural Organization: Some Approaches Based on Network Models and Information Theory. *Annual Review of Neuroscience* 13, 257–281. doi:10.1146/annurev.ne.13.030190.001353

Olshausen, B. A. and Field, D. J. (1996). enEmergence of simple-cell receptive field properties by learning a sparse code for natural images. *Nature* 381, 607–609. doi:10.1038/381607a0

Ortega, P. A. and Braun, D. A. (2013). Thermodynamics as a theory of decision-making with information processing costs. *Proceedings of the Royal Society A: Mathematical, Physical and Engineering Sciences* 469, 20120683. doi:10.1098/rspa.2012.0683. ArXiv:1204.6481 [math]

KEY: ortega$_{t}hermodynamics_{2}013$

ANNOTATION: $Comment : 26pages, 5figures, (underrevisionsinceFebruary2012)$

[Dataset] Sakthivadivel, D. A. R. (2022). Weak Markov Blankets in High-Dimensional, Sparsely-Coupled Random Dynamical Systems. doi:10.48550/arXiv.2207.07620. ArXiv:2207.07620 [math-ph]

KEY: sakthivadivel$_{w}eak_{2}022$

ANNOTATION: $Comment : 17pages.Resultsfromv1generalised.Commentswelcome$

[Dataset] Sanchez, E. H., Serrurier, M., and Ortner, M. (2019). Learning Disentangled Representations via Mutual Information Estimation. doi:10.48550/arXiv.1912.03915. ArXiv:1912.03915 [stat]

Schmidhuber, J. (1991). Curious model-building control systems. *International Joint Conference on Neural Networks*

Tipping, M. (2001). enSparse Bayesian learning and the relevance vector machine. *Microsoft Research* 1. doi:10.1162/15324430152748236

Van Dijk, S. G. and Polani, D. (2013). enINFORMATIONAL CONSTRAINTS-DRIVEN ORGANIZATION IN GOAL-DIRECTED BEHAVIOR. *Advances in Complex Systems* 16, 1350016. doi:10.1142/S0219525913500161
